# Investigating the Impact of Epilepsy on Cognitive Function: A Narrative Review

**DOI:** 10.7759/cureus.41223

**Published:** 2023-06-30

**Authors:** Pranvera Hoxhaj, Sana K Habiya, Rithika Sayabugari, Roghan Balaji, Roshni Xavier, Arghal Ahmad, Mousumi Khanam, Meet Popatbhai Kachhadia, Tirath Patel, Zain U Abdin, Ali Haider, Zahra Nazir

**Affiliations:** 1 Medicine, University of Medicine, Tirana, Tirana, ALB; 2 Obstetrics and Gynaecology, Scher & Kerenyi MDS, New York, USA; 3 Internal Medicine, Indian Institute of Medical Science and Research, Jalna, IND; 4 Public Health, Northeastern Illinois University, Chicago, USA; 5 Internal Medicine, Gandhi Medical College, Hyderabad, IND; 6 Neurology, Ponjesly Super Speciality Hospital, Nagercoil, IND; 7 Neurology, Sri Manakula Vinayagar Medical College and Hospital, Pondicherry, IND; 8 Internal Medicine, Rajagiri Hospital, Aluva, IND; 9 Internal Medicine, Carewell Hospital, Malappuram, IND; 10 Internal Medicine, Ziauddin University, Karachi, PAK; 11 Internal Medicine, Dhaka Medical College, Dhaka, BGD; 12 Internal Medicine, Pandit Dindayal Upadhyay (PDU) College, Civil Hospital Campus, Rajkot, IND; 13 Internal Medicine, American University of Antigua, St John, ATG; 14 Internal Medicine, District Head Quarter Hospital, Faisalabad, PAK; 15 Internal Medicine, Quetta Institute of Medical Sciences, Quetta, PAK; 16 Internal Medicine Clinical Research, California Institute of Behavioral Neurosciences & Psychology, Fairfield, USA

**Keywords:** behavioral problem, neurophysiology. neurology and cognition, epileptic seizures, : epilepsy, cognitive disorders

## Abstract

It has been noted that people who have epilepsy have an increased propensity for cognitive dysfunction. We explored 25 relevant articles on PubMed and Cochrane Library after implementing inclusion criteria. Different factors have been postulated and studied that may cause cognitive dysfunction in these patients; structural brain abnormalities, polypharmacy of antiepileptic medication, and neuropsychiatric disorders are the most common causes. Cognitive assessments such as Montreal Cognitive Assessment (MOCA) and Mini-Mental State Exam (MMSE) are the mainstay tools used to diagnose the degree of cognitive decline, and alterations in EEG (electroencephalogram) parameters have also been noted in people with cognitive decline. The mechanisms and treatments for cognitive decline are still being studied, while attention has also been directed toward preventive and predictive methods. Early detection and treatment of cognitive impairment can help minimize its impact on the patient's quality of life. Regular cognitive assessments are essential for epileptic patients, particularly those on multiple antiepileptic drugs. While proper management of epilepsy and related comorbidities would reduce cognitive decline and improve the overall quality of life for people with epilepsy.

## Introduction and background

Epilepsy is a persistent neurologic condition characterized by clusters of nerve cells, or neurons, which sometimes signal abnormally and cause seizures [1]. Epilepsy, as per International League Against Epilepsy (ILAE), is defined as a disease of the brain characterized by at least two unprovoked seizures occurring more than 24 h apart (or) a single unprovoked seizure with a general recurrence risk of 60% happens in next ten years (or) an identifiable diagnosis of an epileptic syndrome [1-4]. Epilepsy is a disorder; it emphasizes that it comprises many different diseases and conditions. Furthermore, the term disorder implies a functional disturbance, not necessarily lasting, whereas disease may convey a more lasting derangement of normal function [1, 5]. An epileptic seizure is a temporary event characterized by indications or manifestations resulting from atypical, heightened, and coordinated nerve cell activity in the brain. During a seizure, many neurons fire (signal) simultaneously- as many as 500 times per second, much faster than expected. This surge of excessive electrical activity may cause either involuntary movements, sensations, emotions, and behaviors, and the temporary disturbance of regular neuronal activity may cause a loss of awareness [1, 4]. 

Seizures are of heterogenous types but are classified into three large categories - partial (focal), generalized and unknown origin. A partial seizure is a focal or localized event involving specific brain areas, resulting in distinct signs or symptoms. In some focal seizures, the person remains conscious but may experience motor, sensory, or psychic feelings or sensations that can take many forms. In other focal seizures, the person changes consciousness, which can produce a dreamlike experience. Partial seizures may rapidly secondarily generalize and spread to involve all cortical areas. Generalized seizures result from abnormal neuronal activity that rapidly emerges in both hemispheres. These seizures can cause rapid blinking or staring into space, as seen in absence seizures, or more dramatic symptoms, such as loss of consciousness, falling to the ground, and massive muscle contractions, as seen in tonic-clonic seizures. After a tonic-clonic seizure, the individual might feel tired. Structural brain abnormality usually results in focal seizures, and structural, cellular, or biochemical abnormalities involving a more widespread brain region result in generalized seizures. However, there are exceptions in all seizure subtypes [1-8]. 

Approximately 50 million people worldwide have epilepsy [9, 10]. Several studies have consistently shown that the incidence is higher in the older population, especially those over 65 [11, 12]. The point prevalence of active epilepsy is 6.38 per 1000 persons, and the lifetime prevalence is 7.6 per 1000 persons in both rural and urban populations of developed countries [12]. The prevalence of epilepsy did not differ between genders or by age group. Generalized seizures and epilepsy of unknown etiology were most prevalent [9, 10]. Epilepsy has a peak incidence in early childhood, with a second peak occurring for those aged 60 years and older [13]. 

Studies indicate that cognitive dysfunction is more prevalent among elderly individuals with epilepsy, and a significant bidirectional association exists between epilepsy and dementia. Consequently, some people with epilepsy may have an elevated risk of dementia. In contrast, those with particular forms of dementia, particularly Alzheimer's disease and vascular dementia, have a substantially greater likelihood of developing epilepsy [11]. 

Epilepsy is a disorder that affects neuronal networks, and the cognitive and behavioral deficits related to epilepsy are due to the pathological interactions between many brain components. Among the comorbidities associated with epilepsy, cognitive and behavioral abnormalities are the most common and severe. It is widely observed that cognitive comorbidities frequently accompany epilepsy, with the implication being that they are either caused by or a secondary effect of the condition [14-17], Behavior, cognitive, and sleep abnormality significantly contribute to reduced quality of life in patients with epilepsy. Many factors contribute to reduced quality of life, including the etiology of epilepsy; seizure type, frequency, and duration; localization of the epileptic focus; age at onset of epilepsy; physiological and structural changes in the brain secondary to seizures; and adverse effects of antiepileptic drugs (AEDs) [2, 4, 17]. Cognitive and behavioral comorbidities can be both chronic, primarily due to the underlying etiology of epilepsy, and in dynamic evolution because of recurrent seizures or interictal spikes. The type and severity of the impairment are related to the maturational stage of the brain at the time epilepsy appears. Several morphological changes can occur with epilepsy, including cell loss, synaptic reorganization, and changes in neurogenesis. Seizures can also result in physiological alterations in excitatory and inhibitory currents, alterations in the temporal coding of information, and impaired single-cell firing patterns [14, 15, 16]. The disruption of sleep plasticity by epilepsy could result in persistent cognitive impairment, which may be long-lasting or chronic [18]. 

Cognitive impairment is as problems with memory, difficulty with planning, trouble executing tasks, difficulty with language and communication, disorientation to time and place, confusion, difficulty with decision-making and reasoning, problems with focus, concentration, and attention, poor judgment, changes in perception, difficulty understanding, interpreting and navigating the environment, difficulty solving problems, the decline in self-care, changes in personality, mood, and behavior, changes in the coordination and control of body movements, inability to make new memories or learn further information, difficulty recognizing once familiar things, places and people [14, 15]. Cognitive impairment ranges from mild to severe [15]. With mild impairment, people may notice changes in cognitive functions but can perform activities of daily living simultaneously [15, 16]. However, intense levels of impairment can lead to loss of apprehension and impairment in talking and writing resulting in the inability to live independently [14-16]. 

According to recent research, older adults aged 65 years or above with epilepsy experience a faster decline in their global cognitive ability compared to their peers without epilepsy as they age. The erratic and uncertain manifestations of epilepsy can seriously compromise the well-being of individuals affected by the disorder. Recognition of epilepsy as a comorbidity in neurodegenerative diseases is on the rise; however, the extent of its occurrence is often underestimated, and there is a lack of clarity regarding the unique features of epilepsy that manifest in various types of neurodegenerative conditions [19]. The main reason that people with epilepsy don't seek medical attention through either ignorance or lack of awareness of the symptoms. Fear of stigmatization may also cause concealment [9, 20]. 

The treatment landscape for seizures in the United States is vast, with over twenty anti-seizure medications and devices available [21]. Those many options can pose a difficult challenge for healthcare professionals in selecting the most appropriate course of treatment for their patients [21]. Antiepileptic drugs (AEDs) are the first treatment option in patients with epilepsy. Drugs developed after 2000 are known as third-generation antiepileptic drugs [22, 23]. Antiepileptic drugs are chosen based on many factors (age, frequency, type of seizures, associated comorbidities, drugs the patient is currently using, etc.). However, one-third of patients with epilepsy do not improve with AEDs [22, 23]. Another reason for the increased mortality risk is poor compliance and discontinuation of AEDs due to their adverse effects. Therefore, the need for newer AED is high [5, 22, 23]. Monotherapy with a single antiepileptic drug is the most common treatment approach for people with epilepsy. According to current guidelines, carbamazepine or lamotrigine are recommended as first-line treatments for partial-onset seizures, while sodium valproate is preferred for generalized-onset seizures [23, 24]. Broad-spectrum antiepileptic drugs, such as brivaracetam and clobazam, are good choices for generalized tonic-clonic seizures and are well tolerated. Nevertheless, there are various other antiepileptic drug treatments available, and further research is necessary to ascertain their comparative effectiveness, which would aid healthcare providers in selecting the most appropriate course of treatment for their patients [23, 24].

Given the potential impact of cognitive impairment on daily functioning, it is crucial to assess and manage cognitive deficits in individuals with epilepsy. However, detecting cognitive impairment can be challenging, as many individuals with epilepsy may not be aware of their cognitive difficulties. Therefore, it is essential to use appropriate screening tools to identify cognitive deficits early and implement appropriate interventions to manage them. 

We will review the current literature on cognitive impairment in epilepsy, including its prevalence, etiology, and assessment. The paper will also discuss potential interventions for managing cognitive impairment in individuals with epilepsy. 

## Review

Clinical presentation and evaluation of cognitive dysfunction in epilepsy

According to a study conducted by Miller et al. [25], cognitive impairment is common in patients with epilepsy even in the absence of brain lesions, and the number of anti-epileptic medications taken may cause poorer language and visuospatial abilities, as well as higher anxiety-affected visual memory [25]. The study's findings also imply that the combination of epilepsy and other medical diseases linked with age, such as hypertension (HTN) and diabetes mellitus (DM), may raise the risk of cognitive dysfunction [25]. It emphasizes the need to control comorbidities in epileptic patients to reduce the risk of cognitive impairment.

A study showed that subjective cognitive impairment is widespread among individuals with epilepsy, particularly those on polytherapy [26]. In addition, a higher proportion of patients reported cognitive impairment, while a higher proportion of caregivers reported decreased motor coordination [26]. Extensive cognitive assessment should be done in patients taking AEDs, as well as effective management of epilepsy and related comorbidities, which may help to reduce the risk of cognitive impairment and improve the overall quality of life of epileptic patients [26].

Cognitive disturbances are multifaceted and are linked to the reason and age of the beginning of epilepsy, seizure frequency, degree of education attained, and anti-seizure medication (ASM) therapy [27]. 

Patients with treatment-resistant focal epilepsy receiving a pre-surgical evaluation received the most extensive neuropsychological evaluations in epilepsy [27]. The majority of available data are from temporal lobe epilepsy (TLE) and frontal lobe epilepsy (FLE) patients [27]. The location of epileptic seizures and cognitive impairment have been linked in adults [28, 29]. People with temporal lobe epilepsy, for example, have poorer memory [30]. Early-onset epilepsy, particularly epileptic encephalopathies, can also cause severe cognitive impairment [28]. FLE patients have poor executive processes, which include interference and response inhibition, anticipation and planning, verbal and nonverbal motor sequencing, and coordination [31]. 

Evaluating cognitive impairment in people with epilepsy includes education level, duration of epilepsy, age at onset, seizure frequency, and any underlying etiology [32]. Another study found that even newly diagnosed epileptics can have considerable cognitive impairment in areas including memory recall, executive skills, and psychomotor speed compared to healthy people. Furthermore, cognitive impairment, particularly memory impairment, can develop in patients with epilepsy who have no structural abnormalities on MRI, have only a few recorded seizures, and have not yet begun regular anti-epileptic therapy [33].

Detection of cognitive impairment in patients with epilepsy

Cognitive impairment (CI) is a more prevalent comorbidity that affects 70-80% of patients with epilepsy [34]. Early detection and treatment can help to improve the patient's quality of life. Even though it can have a negative impact on the patient's life, it is not well-addressed and researched [35]. The Montreal Cognitive Assessment (MOCA) and the Mini-Mental State Examination (MMSE) are two frequently used screening measures for diagnosing cognitive impairment in epileptic patients [34]. MOCA consists of a 30-point questionnaire that assesses several neurocognitive domains. These are visuospatial/executive, naming, attention, memory, language, abstraction, and orientation (to time and place) [36]. Recent studies suggest that MOCA is a better tool to screen for cognitive decline in epileptic patients due to its high sensitivity, specificity, and accuracy [37]. It is widely used globally as a diagnostic tool in neurology consultations [36]. Each diagnostic tool targets different neurocognitive domains. While the MMSE emphasizes memory, the MOCA focuses on executive function [38]. The MMSE has a ceiling effect or limited dynamic performance range for regular people, making it more likely that people in early cognitive decline will score within the normal range. MOCA has a less ceiling effect [39]. 

Although the MOCA and MMSE are the primary diagnostic tests for detecting cognitive decline in patients, electroencephalography (EEG) studies have been commonly used to detect brain abnormalities. EEG recordings of interictal epileptiform discharges (IEDs) indicate cognitive impairment in epileptic patients [34]. IEDs are the electrophysiological discharges that occur in between seizure episodes thus, are not typically associated with loss of consciousness [35]. Seizure activity can affect cognitive functions while disrupting brain networking, according to a study by Novak et al., [33]. Interictal epileptiform discharges (IEDs), which may be seen in EEG recordings, have also been demonstrated to impact brain activities, particularly memory, significantly [33]. Based on study findings, quantitative EEG can be used to investigate and predict cognitive impairment in patients [40]. Algorithms based on clinical and Phase-locking Value (PLV) EEG data, such as Adaptive Boosting (AdaBoost) and Gradient-Boosted Decision Trees (GBDT), can be effective methods for diagnosing cognitive impairment [34]. 

Along with seizure activity, anti-epileptic drugs (AEDs) are also known to cause cognitive impairment in patients with epilepsy [41]. AEDs impact cognition by limiting neuronal excitability or augmenting inhibitory neurotransmission [42]. Patients treated with AED monotherapy are less likely to experience cognitive decline compared to those treated with polytherapy [33, 43]. Evidence shows that adding each new AED to the treatment regimen raises the risk of cognitive impairment [33]. Neurocognitive functions such as memory, attention, and executive function are frequently affected by AED polytherapy [43]. According to research, newer-generation AEDs have a considerably lower impact on cognitive function than earlier-generation AEDs [33, 44]. According to a study by Quon et al. [41], greater doses of AEDs increase cognitive impairment [41]. Therefore, limiting the number of AEDs to two is recommended to avoid cognitive impairment and to titrate doses carefully [33]. Another study found that the frequency and length of seizures are positively related to cognitive impairment [43]. In one of the studies, 39.5% of epilepsy patients have visual memory impairment, 23.7% have attention and executive function decline, and 15.8% have visual memory and language impairment [25].

Another method used over the last 20 years to assess cognitive impairment (CI) in patients with epilepsy is functional MRI [45]. Resting-state fMRI (rs-fMRI) is a noninvasive approach for detecting variations in brain activity and interregional functional connectivity (FC) by assessing intrinsic blood oxygen level-dependent (BOLD) low-frequency signal fluctuation [46]. Functional connectivity (FC) research employs functional MRI (fMRI) in the resting state or in conjunction with experimental protocols to assess regional brain activation or the degree of dependency between two or more anatomic regions [47]. 

Pathophysiology of cognitive impairment in epilepsy

The mechanisms underlying an increased likelihood of developing cognitive impairment in epileptic patients have not been definitively established. The basal ganglia and thalamus participate in five parallel segregated circuits with selected cortical areas in the frontal lobe [48]. Two of these circuits are related to motor function and influence skeleton-motor and oculomotor areas of the cortex [49]. The remaining three loops relate to non-motor areas in the frontal lobe, including the dorsolateral prefrontal cortex, the lateral orbitofrontal cortex, and the anterior cingulate/medial orbitofrontal cortices [49]. These frontal regions involve planning, working memory, rule-based learning, attention, and emotional regulation, such as the decision threshold in reaction time tasks or the control of automatic visuospatial attention [48, 49].

It is argued that epilepsy is simply one manifestation of the underlying pathological process that might contribute to seizures, cognitive decline, psychological problems, systemic illness, and, perhaps indirectly, psychosocial difficulties [50, 51]. Seizures are seen in the prodromal phase of several neurodegenerative illnesses, and some studies in patients with mild cognitive impairment have reported that cognitive decline may begin several years earlier in those individuals who suffer from seizures compared to those who do not [11, 50, 51].

The pathophysiology behind the mechanisms of the relationship between epilepsy and cognition is that the seizure and epileptiform discharges in the EEG directly injure neural networks, which provide a stepping stone for cognitive impairment. Nonetheless, the action potential is the fundamental unit of information processing in the brain, and sequences of action potential firing in neuronal populations over time are therefore considered mechanisms of cognition, as cognitive function is explicitly about information processing. Furthermore, frequent seizures, especially status epilepticus, repeatedly cause oxidative stress and neuronal loss, which is finally associated with cognitive impairments [52, 53]. 

Studies of whole brain volumes have revealed associations between cognitive impairment and brain volume losses in patients with epilepsy [46]. In adult patients with TLE, those with early age at epilepsy onset had a reduction of total brain volume, especially of white matter, compared to patients with late age at epilepsy onset and healthy controls. These total white matter volume reductions were correlated with poorer cognitive performance across multiple cognitive domains, including verbal fluency, spatial orientation, verbal and nonverbal memory, and response inhibition. In addition, these patients had lower baseline volumes of gray and white matter, smaller left hippocampal volume, more significant baseline cerebrospinal fluid (CSF) volume, and lower intellectual capacity. Atrophy of the thalamus, amygdala, and mammillary bodies is associated with impaired memory performance and language impairment in patients with epilepsy [26, 46, 53-58].

Recent studies have linked individual tract integrity to cognitive dysfunction in epileptic patients [59-62]. For example, the integrity of the left uncinate fasciculus was correlated with verbal memory performances in patients with left TLE. Other studies demonstrated that cognitive performances correlated with the integrity of multiple cortical-to-cortical association tracts and limbic projection tracts, especially in the left hemisphere [59-65].

Seizures, causes of seizures, interictal spikes (IIS), and AEDs all significantly impact the development of cognitive impairment in epileptic patients [28,66]. A decline in spatial performance is noted in patients with recurrent seizures. Recurrent seizures are known to impair long-term potentiation (LTP) and the power and frequency of theta oscillations that indicate the orderly communication of networks. These factors may have an adverse impact on cognition [28].

**Figure 1:**
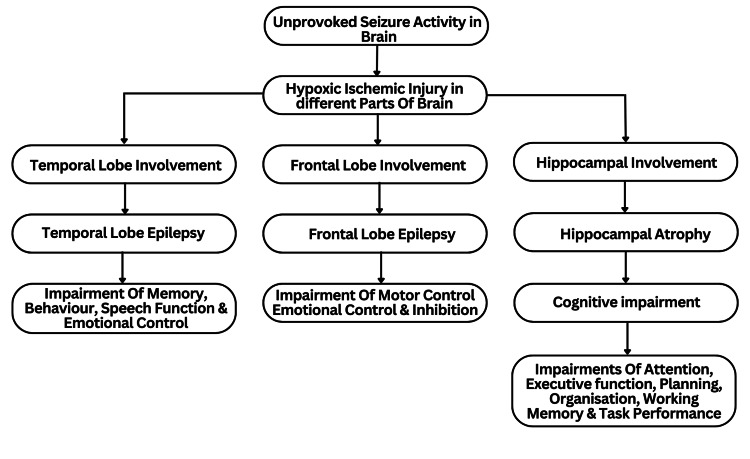
Pathophysiology of seizure causing memory impairment Source: reference no. [26]

Frequency encoding, rate coding, and population coding are the mechanisms associated with neuronal firing and information encoding. In rate coding, the information is carried by the number of action potentials that each neuron fires. In population coding, information is transmitted through the coordinated firing of a group of neurons. The process by which the timing of action potential firing, in the order of milliseconds, encodes information is known as temporal coding. Disruption of these coding processes is noted in patients with epilepsy [66]. The power of gamma and other oscillations in the hippocampus is reduced due to the inhibitory wave that occurs after IIS. During Status epilepticus, increased excitation and decreased inhibition lead to changes in the brain. These changes result in abnormal neural circuitry, which in turn leads to the development of cognitive impairment [28]. The pathophysiology has been further summarized in Figure 1.

Treatment possibilities for epilepsy-induced cognitive impairment

Despite the increasing incidence of cognitive dysfunction among epilepsy patients, currently, available therapies to treat CI in epilepsy are limited [67]. Treatment for cognitive deficits in patients with epilepsy is individualized, based on identifying the contributing factors [33]. Cognitive deficits in epilepsy are usually managed indirectly by aggressive seizure control (including earlier epilepsy surgery), selecting antiepileptics with good cognitive profiles, and treating comorbid conditions such as depression [68]. The beneficial effects of antiepileptics in reducing seizures may offset their adverse cognitive effects. Furthermore, there is limited evidence of the effectiveness of AEDs, vagal nerve stimulation (VNS), transcranial magnetic stimulation (TMS), and behavioral strategies [69].

Antiepileptic Drugs Influence on CI

Numerous studies have found that people with epilepsy who take more AEDs have a higher risk of cognitive impairment [25, 33, 46]. Patients taking AEDs, like any pharmacological medicines, develop more pronounced adverse effects when combining more than one AED or while taking greater doses of the drugs [33]. The outcomes of one study show that each additional medicine impacts the cognitive side effects of antiepileptic pharmacotherapy [70]. Therefore, antiepileptic polytherapy with more than two concurrent drugs should be avoided. A combination of AEDs with good cognitive qualities may lower the incidence of drug-load-related side effects [70].

Furthermore, except for topiramate, studies demonstrate that older generations of AEDs (e.g., carbamazepine, valproate, and phenytoin) produce much more adverse effects and negatively influence cognitive functioning than newer groups [71]. The first problem is the inability to split the treatment regimen at random because people with more resistant epilepsy are likelier to try newer medications, and the impact on their cognitive abilities is not indicative of the broader population with epilepsy [33]. Conversely, successful therapy lessens the severity and frequency of seizures and partially eliminates the harmful consequences of seizures on cognitive capacities [72]. Secondly, another factor is the difficulty in determining comparable doses of drugs because the doses are individually adjusted. Different doses may have different effects on cognitive function [33]. 

The Effect of Vagal Nerve Stimulation on Epilepsy

Transcutaneous auricular vagus nerve stimulation (taVNS) is a noninvasive neurostimulation method that stimulates the vagus nerve's auricular branch outside the ear [73, 74]. Vagal nerve stimulation is one of the treatments for refractory epilepsy that can help with depression and cognitive issues. Both of these effects may aid in the reduction of cognitive impairment in epilepsy patients [68]. Noninvasive neurostimulation techniques have recently acquired popularity, aiming to induce a neuromodulatory effect without an intrusive operation. Individuals with cognitive impairments had a reduced effect on seizure frequency in one trial. However, most individuals in the cognitive deficits group got at least one benefit from treatment - seizure reduction or other improvements. The findings of this study imply that seizure frequency reduction should not be the main criterion for VNS therapeutic effectiveness and that patients with cognitive abnormalities may benefit from the treatment to some extent [69]. 

Another study of 20 subjects that looked at the effects of VNS on attention, cognition, and emotional reactivity discovered that VNS increased working memory performance [75]. A more extensive trial of 112 individuals who received VNS therapy demonstrated improved quality of life but no meaningful improvement in depression-related measures [76]. Klinkenberg et al. [77], on the other hand, performed a six-month follow-up prospective longitudinal observational cohort analysis of 41 patients with intractable epilepsy treated with VNS and showed that patients had lower levels of anxiety, tension, and melancholy [77]. The existing research is equivocal, although it primarily supports VNS's favorable effects on mood.

Transcranial Magnetic Stimulation (TMS)

TMS stimulates nerve cells by using a magnetic field in the brain to improve the symptoms of epilepsy. With TMS, a large electromagnetic coil is placed against the scalp. The electromagnet creates electrical currents that stimulate nerve cells in the region of the brain involved in epilepsy [78]. It is also a noninvasive brain stimulation treatment that can potentially improve memory. Mild cognitive impairment (MCI), for which no specific treatment exists, is a clinical state associated with an elevated risk of dementia; TMS aids in its treatment [79]. Repetitive transcranial magnetic stimulation (rTMS) is an emerging treatment for cognitive impairment caused by epilepsy, a common neurological disorder in which many people do not respond adequately to treatments [78]. Drug-resistant epilepsy is associated with a lower quality of life, and such patients are frequently subjected to surgery or other invasive procedures, both of which involve considerable risks. In addition, even those who respond well to pharmacological therapy may struggle with the potential side effects of their drugs. rTMS, on the other hand, is a painless, non-invasive treatment that, if practical, has considerable advantages over both antiepileptic medications and surgical treatments [79].

Behavioral approaches are used in individuals with epilepsy alone and in combination and as an adjuvant to medicine [72]. The mechanism of potential seizure frequency reduction is unknown. Still, it could be related to reduced stress and associated maladaptive responses, reduced comorbid psychiatric symptoms, increased self-efficacy to improve seizure management, or learned behaviors that alter the underlying epileptogenic neural networks. CBT for treating cognitive impairment in epilepsy may be successful due to lifestyle modifications that lessen the influence of seizure triggers or improve treatment adherence, as well as a direct association between cognition and seizure activity. Tang et al. [80], proposed a model in which the long-term practice of psychobehavioral strategies to reduce seizure precipitants ultimately leads to changes in neuronal circuits and a decrease in the epileptic disposition [80].

More attention and awareness measures are required among healthcare professionals regarding epilepsy and cognitive impairment. Proper epilepsy and comorbidity management may reduce the risk of cognitive deterioration and improve the overall quality of life in people with epilepsy. Furthermore, treating cognitive deficiencies in epilepsy demands a comprehensive and tailored method. More extensive research is needed to understand the efficacy of different treatment methods better, identify specific patient subgroups that may benefit the most, and discover potential synergies between these techniques. By expanding our understanding and therapy options, researchers may be able to improve the cognitive well-being and quality of life of persons with epilepsy.

There are significant gaps in knowledge regarding the effect of epilepsy on cognitive impairment and vice versa. Accurate estimates are needed to inform public health policy and prevention and understand these populations' health resource needs. Although our review has been conducted on an important topic, our study had limitations. Our review of these findings concludes that although current data do not allow us to make definitive inferences, they show that this is an important area for an investigation that has potential application to patients with epilepsy and cognitive impairment. The main limitation of most current clinical research is the small sample of subjects. This calls for more investigations with patients to indicate better how epilepsy affects cognitive functions and which treatments can help ease those symptoms. In addition, more studies are needed to determine what other risk factors can affect cognitive functions in these patients. Our review also underscores the need for further research to explore the most appropriate treatments for cognitive impairment in patients with epilepsy. Another area for improvement is that we did not include any statistical analysis. However, it is one of the few articles reviewing the impact of epilepsy on cognitive function and discussing the possible treatments for cognitive impairment.

## Conclusions

It is observed that cognitive impairment frequently accompanies epilepsy, and they are more common in patients with epilepsy than in the general population. Therefore, the diagnosis and appropriate management of cognitive impairment in epileptic patients are crucial steps in providing the most efficient medical care for both the patient and the overall community. There is currently no consensus on a diagnostic definition for cognitive disorders in epilepsy. Electrophysiological and neuroimaging investigations in epilepsy have focused interest on network-level dysfunctions. 

In summary, this narrative review underscores the detrimental impact of epilepsy on cognitive function. The findings emphasize the importance of comprehensive care for individuals with epilepsy, addressing both the seizure control and cognitive management aspects. By increasing our understanding of the relationship between epilepsy and cognitive function, we can improve the quality of life and outcomes for individuals living with this condition.
